# Where Is It Like to Be an Octopus?

**DOI:** 10.3389/fnsys.2022.840022

**Published:** 2022-03-14

**Authors:** Sidney Carls-Diamante

**Affiliations:** Zukunftskolleg/Philosophy Department, University of Konstanz, Konstanz, Germany

**Keywords:** octopus consciousness, octopus arm, octo-munculus, multiple consciousness, unity of consciousness

## Abstract

The cognitive capacities and behavioural repertoire of octopuses have led to speculation that these animals may possess consciousness. However, the nervous system of octopuses is radically different from those typically associated with conscious experience: rather than being centralised and profoundly integrated, the octopus nervous system is distributed into components with considerable functional autonomy from each other. Of particular note is the arm nervous system: when severed, octopus arms still exhibit behaviours that are nearly identical to those exhibited when the animal is intact. Given these factors, there is reason to speculate that if octopuses do possess consciousness, it may be of a form highly dissimilar to familiar models. In particular, it may be that the octopus arm is capable of supporting an idiosyncratic field of consciousness. As such, in addition to the likelihood that there is something it is like to be an octopus, there may also be something it is like to be an octopus *arm*. This manuscript explores this possibility.

## Introduction

It has recently been suggested that the octopus possesses “two brains” (Grasso, 2014). In particular, these are the central brain and the *brachial plexus*, or the network formed by the interconnection of *axial nerve cords*, of which every arm has one. As will be discussed in detail shortly, the axial nerve cords are considered high-level neural centres within each arm, due to their processing and control responsibilities (Richter et al., 2015). The complexity of the octopus’s arm nervous system—which makes up the bulk of the peripheral nervous system (PNS)—is such that each arm demonstrates organisation “like the brain of a living organism…with a diversity of sensory modalities, motor neurons effecting different motor systems and large central neuropils which are processing centres for large amounts of information” (Grasso, 2014, p. 103). Such features are what prompted suggestions that octopus arms may house local “brains.”

Although the brain and arm nervous system are dissimilar in their functions and structure, both make extensive and non-redundant contributions to cognition and behaviour in octopuses. In order to describe the complex interplay between the central and peripheral components of the octopus neuro-cognitive system, Grasso (2014) uses the metaphor of an “octo-munculus” as an illustration. This octo-munculus would be “a brain-to-body spatial map…(like the human ‘Homunculus’)…depicted as information processing systems distributed throughout each arm and a brachial centre in the brain” (Shigeno et al., 2018, p. 11).

Now, since philosophy has a long history of associating brains—or in this case sophisticated neural structures with considerable functional autonomy and anatomical demarcation—with minds or consciousness, it is not unreasonable to wonder about what kind of subjective, phenomenal experience would arise from such a nervous system as that of octopuses. Indeed, the recent years have seen an increase in interest in consciousness in octopuses. Since these animals exhibit behaviour deemed to be indicative of consciousness, yet have nervous systems that are greatly dissimilar from those associated with the capacity to support consciousness, octopuses proffer the possibility of a radically different form of consciousness from what we are currently familiar with. In particular, due to their highly distributed neurocognitive systems with highly autonomous components, the possibility has been raised that octopus consciousness might consist of multiple conscious fields that may or may not be experienced as a single, unified field. This question remains open, and for now I must postpone an attempt at addressing it.

Nevertheless, the very features of the octopus nervous system that suggested disunified consciousness present another potential way wherein octopus consciousness differs from familiar models: octopuses may house two different types of consciousness, with dissimilar complexity, contents, functions, and contributions to cognition. Thus, in addition to speculating about “What it is like to be an octopus?” (see Nagel, 1974), one might also ask “*Where* is it like to be an octopus?”. This manuscript explores this latter question, by *raising the possibility* that octopus arms have their own respective conscious fields. In order to achieve this aim, I present a number of principled reasons to surmise about the presence of “arm-based” consciousness in octopuses.

The manuscript proceeds as follows. Section “Consciousness” discusses the construals of consciousness utilised for present purposes. Section “Octopus Nervous System” provides a description of the octopus nervous system and features that are of particular interest. Section “Attributing Consciousness to Octopuses” surveys the bases for consciousness attribution in octopuses. Section “Arm-Based Consciousness” presents the principled reasons for speculating about arm-based conscious fields. Finally, section “Concluding Remarks” concludes the manuscript.

## Consciousness

Consciousness is often used equivocally to refer to several related but dissimilar capacities. In this manuscript, I understand consciousness as the set of phenomenal states experienced simultaneously at any given point in time, which are accessible to the neurocognitive system for use in cognitive processes such as the control of behaviour (Block, 1995; Baars, 2005). Consciousness here is synonymous with phenomenal experience, in that “there is something it is like” to be the conscious organism in question (Nagel, 1974). In its most rudimentary form, consciousness consists of perceptual or sensory experience evoked by external and internal stimuli. These include awareness of one’s external surroundings and internal states that have phenomenal qualities (in contrast to those that are not “felt”, such as blood circulation or digestion). As such, consciousness has both exteroceptive and interoceptive sensory contents.

The literature on consciousness distinguishes between *primary* and *higher-order consciousness*, based on the complexity of its contents. Primary consciousness is that kind of consciousness sometimes equated with the capacity for sensory awareness. For an organism to have primary consciousness, all it needs is the capacity for “direct awareness of the world without further reflection upon that awareness” (Barron and Klein, 2016, p. 4901). Insofar as consciousness attribution is concerned, it is believed that primary consciousness is widespread throughout the animal kingdom. In contrast, higher-order consciousness involves capacities for metacognition that vary in complexity (Seth, 2009). An example of these metacognitive capacities is consciousness of being conscious, i.e., awareness that one is experiencing the conscious states that one is experiencing. Another complex manifestation of higher-order consciousness is a sense of self, wherein the organism recognizes itself as the subject that experiences the experiences it has. It is also believed that more sophisticated forms of higher-order consciousness subserve the ability to mentally “construct past and future scenes” (Seth, 2009, p. 9); a capacity for both short- and long-term memory is thus presupposed in higher-order consciousness.

Another important distinction pertains to the structure of conscious experience. Bayne (2010) distinguishes between a *conscious field* and a *conscious stream.* The former is the cluster of conscious states experienced simultaneously at any single time, while the latter is the series of conscious states experienced over time. It has long been assumed that the “normal” structure of consciousness is that it is *unified*, in that a conscious organism experiences a single conscious field at any given time (Bayne, 2008, 2010). This notion has been putatively challenged by phenomena such as the split-brain syndrome, wherein the corpus callosum is severed (originally to prevent the spread of epilepsy across brain hemispheres). Apparent mostly in experimental settings, the split-brain syndrome involves “information presented in the [right visual field being] unavailable for left-handed grasping behaviour while information presented in the [left visual field is] unavailable for verbal report” (Bayne, 2010, p. 192). Octopuses have appeared to be another challenger to the unity thesis, because of the extensive distribution of their nervous systems and cognitive routines and the considerable autonomy displayed by the highly elaborated peripheral nervous system. While there is accumulating evidence in favor of unity (Mather, 2021), adjudicating whether octopuses experience multiple conscious fields or a single one requires independent investigation outside of present purposes.

The discussion on consciousness will proceed following an overview of what the octopus nervous system is like.

## Octopus Nervous System

### Anatomy and Functions

With its 500 million neurons—a number more typical of vertebrates such as dogs—octopuses have the largest nervous systems among invertebrates (Hochner, 2004). The octopus nervous system is highly distributed, and typically divided along anatomical lines into components with considerable functional autonomy. The three main parts of the octopus nervous system are the *brain*, the *optic lobes*, and the highly elaborated *arm nervous system*. Significantly, the arm nervous system contains three-fifths of the octopus’s neurons. Importantly, the brain, optic lobes, and arm nervous system are interconnected by only about 30,000 nerve fibres, suggesting that “much of the processing of motor and sensory information is performed in the peripheral nervous system and the optic lobes” (Hochner, 2012, p. R889).

The central nervous system (CNS) consists of the brain, which with 45–50 million neurons is the smallest component of the nervous system. The brain is responsible for integrating information received from the different parts of the nervous system, as well as high-level “cognitive and executive functions like motor coordination, decisionmaking (*sic*), and learning and memory” (Levy et al., 2017, p. 7). For instance, the brain is responsible for selecting and initiating or terminating a particular behaviour or action, but the details required for realising arm movements are embedded within the arm nervous system (Sumbre et al., 2005, 2006). The brain contains the basal lobes, the highest motor control centres in the octopus. Early on, it was discovered that stimulating different parts of the basal lobe evokes different types of complex movements: “electrical stimulation of the anterior basal lobe…produces effective walking movements of the arms, stimulation of the median basal produces swimming, and of the lateral basal changes in colour over the whole skin” (Young, 1971, p. 14). The vertical lobe system, which processes visual memory and are vital for cross-brain transfer of visual information, is also found in the brain. When the vertical lobes are removed, memory transfer is impaired, in direct proportion to the extent of excision.

The paired and lateralised optic lobes are usually considered part of the peripheral nervous system (PNS), but are sometimes regarded as part of the CNS. Between them, the optic lobes have 120–180 million neurons. Each optic lobe is responsible for processing visual information received *via* the ipsilateral eye. Octopus eyes are highly developed and comparable to those of vertebrates. Octopuses are highly visual, especially when it comes to navigation and learning. They have lateralised vision, and are able to use a single eye for perceptual and learning tasks. Signals received *via* one eye are transmitted and processed in its ipsilateral optic lobe, which sends this information further upstream for “cross-brain transfer” (Mather, 2021, p. 408). Tasks learned while using one eye can later be performed with the other eye, although not as accurately or efficiently as with the original one. This is because “cross-brain transfer [of information from one eye] would normally be complete but storage or retrieval would not be as good in the contralateral as in the ipsilateral area of the brain” (Mather, 2021, p. 408).

The most notable—and largest—component of the PNS is the arm nervous system, in which 350 million neurons are distributed equally between the eight functionally and anatomically^1^ identical arms. The arms are interconnected to each other and to the brain by a ring of fibres at their bases, often referred to as the *interbrachial commissure.* Within each arm can be found an *axial nerve cord*, four *intramuscular nerve cords, sucker ganglia*, and millions of *sensory receptors* responsive to chemical, tactile, mechanical, and proprioceptive stimulation. The axial nerve cord is a high-level sensory processing and motor control centre, which integrates information from the respective arm with the commands it receives from the CNS (Richter et al., 2015). The intramuscular nerve cords play an important role in the motor control of the arm. They receive proprioceptive information from proprioceptors in the arm, which are embedded outside the *suckers* (Grasso, 2014). On the underside of each arm are numerous highly sensitive suckers arranged in a double row. With thousands of sensory receptors and motor neurons each, suckers are an important source of tactile, chemical, and spatial information (Grasso, 2014). They are used in a great variety of octopus behaviour, such as object manipulation and locomotion, for instance “walking” along a surface (Grasso, 2014). Each sucker is innervated by its own sucker ganglion, which does not communicate directly with other sucker ganglia (Grasso, 2014). Instead, information from one sucker is channelled *via* the axial nerve cord to nearby ones.

### Notable Features

There are two features of the octopus nervous system that stand out as being unique and unusual. The first is the brain’s inability to support *somatotopic representation* or point-for-point mapping of the body, and the second is the extensive autonomy in sensory processing and motor control of the arm nervous system. These will now be discussed in turn.

Following stimulation experiments to the basal lobes, which are the octopus’s highest motor control centres, it was discovered that the octopus brain is incapable of somatotopically representing the body (Zullo et al., 2009). Rather than a somatotopic map, it is likely that what are represented in the octopus brain are motor programmes or functions (Zullo et al., 2009), which can then be consolidated with sensory information sourced from all over the nervous system (Zullo and Hochner, 2011). The absence of a somatotopic map was inferred following findings that direct stimulation to the basal lobes led to identical movements of multiple arms whereas generating movement in a specific arm was not possible, and that the same pattern of movement can be evoked by stimulating different parts within the basal lobes. What these findings imply is that motor commands from the brain are global, and received by multiple, if not all, arms instead of by a particular appendage; it is hypothesised that the brain “generates only one motor command to all arms if they are activated in the same behavioural context” (Levy et al., 2017, p. 12). It may thus be the case that the brain is incapable of proprioceptively distinguishing between individual arms, or if it were, it might not do so robustly. Activation of a single arm for use in a task, such as reaching, would then require extensive participation of the PNS and cannot be accomplished by the brain alone.

Moreover, these findings are in line with the fact that the neural resources of the octopus brain are inadequate to “be able to deal with such a huge number of parameters that would be sufficient to represent its muscular system” (Levy et al., 2017, p. 3). A rigid skeleton would have supplied permanent structures to serve as proprioceptive landmarks that would facilitate somatotopic representation and motor control (Wolpert, 1997; Gutfreund et al., 1996). However, octopuses lack a skeleton, and furthermore have soft bodies with arms that are “unsegmented…and can deform at any point along their length. Each arm can, at any point along its length, bend in any direction, elongate, shorten, and twist either clockwise or counterclockwise” (Levy et al., 2017, p. 3). The demands of somatotopically representing such a body are exorbitant, and have been proposed by some authors to be beyond of any biological system (Levy et al., 2017). As compensation, the octopus evolved a unique solution to the demands both of monitoring and controlling such a flexible body with countless movement possibilities and processing integrating multi-modal information from its millions upon millions of sensory receptors: the development of a highly elaborated and autonomous PNS.

It has been said of the arm nervous system that it appears to be “in some ways curiously divorced from the rest of the brain and many of the arms’ actions are performed without reference to the brain” (Hanlon and Messenger, 1996, p. 15). The extent of such independence from the brain was most dramatically demonstrated in early studies on severed octopus arms (Rowell, 1963). In these studies, it was discovered that touching the suckers evoked the grasping reflex, for up to three hours after the arm had been amputated, thus proving that sucker control was localised within the suckers and their respective ganglia. In the same vein, it was discovered that when pricked, a freshly amputated arm would demonstrate a number of responses identical to those found in intact animals. The first set of findings showed that the suckers would grasp at whatever surfaces they came into contact with, and that the grasping was “stronger than that normally elicited from the intact animal” (Rowell, 1963, p. 259). These findings indicated that the control of suckers is mainly localised within their respective ganglia: the brain may “influence but…not specify the detail of ongoing sucker and arm behaviour” (Grasso, 2014, p. 114). The second set of findings demonstrated that when pricked with a needle, the arm generates the following responses: “a local flinching of the skin, due to contraction of the dermal and subdermal muscle layers, a movement of the whole arm withdrawing it from the stimulus, and flexion of the arm and protrusion of the suckers in a way likely to cause them to come into contact with the stimulating object” (Rowell, 1963, p. 259–260).

In the same vein, amputated or neurally isolated arms are able to produce movements in response to electrical or tactile stimulation that are almost identical to those in intact octopuses (Sumbre et al., 2001). These findings demonstrate that “the basic motor programme for voluntary movement is embedded within the neural circuitry of the arm itself” (Sumbre et al., 2001, p. 1845). Thus, whereas selecting and activating motor programmes is the responsibility of the brain, the actual “instructions” for bringing the arm into the required shape are contained within the arm. Since the muscles of octopus arms are arranged *hydrostatically*, wherein contraction in one muscle group produces compensatory lengthening in the others (Kier and Smith, 1985), and do not contain any fixed structures, they have potentially unlimited degrees of freedom (DOFs) of movement. However, in order to simplify motor control, octopuses have evolved a set of *stereotypic motor* programmes (Sumbre et al., 2001, 2006) that are used in the majority of its actions. Thus, rather than formulating commands to bring the arm muscles into the required shape from scratch every time, motor control labour is reduced to orienting the arm correctly and scaling the velocity of the movement (Gutfreund et al., 2006). In addition to simplifying motor control demands, the use of stereotypic motor programmes also dissolves the need for somatotopic representation in the motor centres in the brain.

It has been proposed that octopus arms are capable of somatotopic mapping (Grasso, 2014). To understand how, we must follow Grasso’s (2014) deconstruction of the octopus arm into local brachial modules (LBM), which “contain the neural components of each sucker-ganglion/brachial-ganglion pair (i.e., the primary receptors [chemo-, mechano-, and proprioceptive], the motor neurons and the interneurons)” (p. 102). Now, each LBM is provided with sensory information by its respective suckers. Importantly, the rims of the suckers “necessarily form a topologically ordered spatial array” (Grasso, 2014, p. 105). This is because the close double-row arrangement of suckers along the arm entails that each sucker will come into contact with the same object or surface at different locations. Since each sucker transmits information to its own ganglion, this information is “location-specific”: each sucker ganglion receives information about a different area of the object or surface in question, which the higher processing centres receiving this input are able to consolidate into a more holistic “picture.” Importantly, the activation patterns produced by sucker activity and the movements of the arm that accompany it are “stored and remembered hierarchically *across the network of ganglia* [and] have an ordered spatial arrangement that reflects the attitude of the animal’s body and state of the external world as sensed by contact with surfaces” (Grasso, 2014, p. 110). Furthermore, these activation patterns also reflect the temporal sequence in which they occurred. Since the arm moves in order to bring the suckers into contact with the object, some suckers are bound to touch it before others. This entails that the corresponding activation by the LBMs they belong to occurs before others, therefore allowing the arm nervous system to monitor the movement of the arm in question. Additionally, information about the activity of the arm and its suckers may be stored for minutes or possibly even up to an hour and recruited for use in learning, suggesting that the arm nervous system is capable of memory and perhaps even representation (Grasso, 2014). Thus, due to the intrinsic topographical and temporal organisation of information received *via* the sensory and mechanoreceptors within the arm, there is a possibility that in contrast to the brain, “a somatotopic map might be formed by the arm” (Grasso, 2014, p. 115) of that arm.

## Attributing Consciousness to Octopuses

### Neuroanatomy and Neurophysiology

In the Cambridge Declaration on Consciousness (Low et al., 2012), octopuses were declared part of the list of candidates for consciousness on the basis of having the “neuroanatomical, neurochemical, and neurophysiological substrates of conscious states along with the capacity to exhibit intentional behaviours” (p. 2). Significantly, octopuses possess brain structures analogous or homologous to those in vertebrates (Shigeno et al., 2018) that are associated with consciousness. Taken together, these structures and their functions in the octopus suggest that if they are, like their vertebrate counterparts, involved in generating CNS-based consciousness, the resulting phenomenal field may be fed by information of multiple modalities from all over the nervous system.

An important structure for consciousness in vertebrates is the thalamus, which receives much of the information that is headed for the cerebral cortex. The thalamus then “transmits this information and…receives an even greater number of reciprocal connections back from the cortex” (Blumenfeld, 2016, p. 8). As such, the contents of consciousness are relayed *via* the thalamus (Blumenfeld, 2016). An analogous structure in cephalopods might be the dorsal basal and sub-vertical lobes, as they “receive many input fibres from the entire body *via* direct and indirect pathways from the sub-oesophageal mass, suggesting that it is a relay centre from the “cortically located” frontal and vertical lobes in cephalopod brain (*sic*),” (Shigeno et al., 2018, p. 8). In addition to these structures, the inferior frontal lobe is another potential analogue to the thalamus, being “a major chemo-tactile sensory-motor centre processing information originating from the suckers and arms” that is “is involved in learning and memory recall being part of the so-called chemo-tactile memory system” (Shigeno et al., 2018, p. 8). In its processing functions, it resembles the vertebrate olfactory cortex.

It is believed that the vertical lobe system has deep homology with the cerebral cortex (Shigeno et al., 2018), whose responsibilities include “regulating the overall level of consciousness” (Blumenfeld, 2016, p. 16). The basis of this hypothesis is that certain behaviours, in particular “sleeping, decision-making, discrimination learning and lateralisation of the brain” (Shigeno et al., 2018, p. 9) exhibited by some cephalopods such as octopuses are may be indicators of advanced cognitive capacities. Since in mammals, at least, such behaviours require a cerebral cortex, the presence of an equivalent structure in cephalopods must be inferred. The vertical lobe system is involved in the processing of tactile and visual memories, and when removed “impairs long-term memory for new tasks” (Hochner, 2004, p. 4). In its roles in memory in learning, the vertical lobe is similar to the hippocampus in vertebrates (Hochner, 2004). It has also been discovered in early studies that the vertical lobe “is somehow connected with restraint…and [might serve] to introduce into the system the effect of nerve fibres [from the arms and mantle] that signal trauma (pain)” (Young, 1971, p. 244). Furthermore, together with the subvertical lobe, the vertical lobe may “amplify such pain signals, in the sense of putting them into more channels, and to insert them in such a way that they have an appropriate effect on the system” (Young, 1971, p. 244).

### Behaviour

The sophisticated and complex behavioural repertoire of octopuses is likewise notable because it is the outcome of *domain-general cognition* (Vitti, 2013). In contrast to *domain-specificity*, wherein cognitive capacities are limited to those that are immediately required to survive within a particular ecological niche, domain-generality recruits multiple cognitive domains and thus produces cognitive abilities and behaviour that are flexible and adaptive within a wide variety of situations. Since domain-generality is facilitated by a centralised organisation of the nervous system, it is typically associated with vertebrates, who have highly centralised neurocognitive systems. Furthermore, the emergence of domain-general cognition is believed to have been influenced by sociality, which demands the capacity for adaptive responses to conspecifics and non-conspecifics alike. As such, it is somewhat surprising that octopuses, with their decentralised and distributed nervous systems, and largely solitary life styles would exhibit such behavioural capacities.

However, although modern octopuses are for the most part solitary, their evolutionary history reveals the heavy ecological demands that would have encouraged the emergence of sophisticated cognition and adaptive behaviour. Known as the Packard scenario (Packard, 1972), after its proponent Andrew Packard, the predominant theory is that due to the internalisation and reduction of the ancestral shell—a feature of all coleoid cephalopods, but none more extensive than in octopuses—octopuses lost the capacity for buoyancy. They consequently sank down to the benthos or sea floor—to this day is their natural habitat—which is rich in ecological diversity. In order to survive, octopuses would have had to learn how to interact adaptively with a large number of fellow benthic species—many of which were vertebrates—predator and prey alike (Borrelli and Fiorito, 2008). These ecological pressures thus set the stage for the development of their cognitive and behavioural sophistication, much of which has attracted the attention of researchers. This section presents a number of examples of octopus behaviour that suggest the presence of consciousness.

Octopuses have demonstrated capacities for learning, with regards to behaviour and discriminatory tasks. For instance, when handling unyielding bivalve prey, they employ different techniques selected through trial and error until they are able to get at the edible portions (Mather, 2008). This stands in contrast to perseverating with an ineffective technique, which suggests lower cognitive flexibility. Octopuses are also known for unpredictability and plasticity, rather than fixed or stereotyped responses, in their avoidance behaviours in the face of stimuli previously experienced as negative (Mather, 2008). Similar to vertebrates, octopuses are capable of associative and reverse associative learning, sensitisation and habituation to stimuli, using multiple cues in visual discrimination tasks, stimulus generalisation, spatial learning, and conditional discrimination. They have also demonstrated capacities to learn about objects not encountered in the wild, in the form of different types of sensory discriminations. They can visually distinguish between orientations, rotations, and mirror images, as well as tactilely discriminate between shapes, curvature, and striation of objects not encountered in the wild (Wells, 1964; Wells and Wells, 1957). Taken together, these learned discriminations suggest that octopuses may be capable of concept formation (Mather, 2008).

Another important capacity that subserves consciousness is memory. Storage and retrieval of information stored in memory decouples the organism from the environment, as this information remains accessible over time, without requiring the presence of the original stimuli or scenario. Memory also allows the mental reconstruction of past scenes, a task held to be achievable only with the involvement of consciousness; furthermore, where a capacity for planning is present, memory provides information that can be recruited for use in mentally constructing future scenarios and formulating actions needed to bring or avoid certain states of affairs. Octopuses are capable of storing short- and long-term memories, the latter of which can stay stable for months (Hochner et al., 2006)—which is remarkable since their typical life spans are 1–2 years.

Octopuses’ capacities for memory are also highlighted in their use and occupancy of dens. Denning behaviour is exhibited by many octopus species, wherein a hole is dug in the seabed or any other soft substrate, and used as a residence for several days to a few weeks. In some cases, octopuses collect stones and arrange them around the opening of the den. Octopuses usually capture prey by going on hunting trips that can last up to several hours and cover large distances, after which they return to the den with the prey to eat. Significantly, they do not use fixed or predictable routes when leaving and returning to the den (Mather, 1991). Furthermore, it has been discovered that octopuses use prominent physical features of the environment as navigational landmarks (Mather, 1991; Hvorecny et al., 2007). In addition to demonstrating the use of memory, denning behaviour is further suggestive of a number of advanced cognitive capacities predominantly observed in vertebrates. Among these are the ability to form mental maps of areas surrounding their dens (Hanlon and Messenger, 1996), the capacity for concept formation manifested as being able to recognize a given feature of the environment from different angles, and conditional discrimination or the ability to “discriminate between potential cues [present in the environment] and show context (condition) sensitivity” (Hvorecny et al., 2007, p. 449). In the context of navigating using environmental landmarks, conditional discrimination is expressed as identifying a certain feature as distinct from similar ones and determining its “significance” in the given context.

Octopuses may also have a sense of self, rudimentary manifestations of which include awareness of one’s own physical boundaries that demarcate one from the external world (see also Merker (2005), Godfrey-Smith (2013)), and the capacity to distinguish between oneself and another organism. It has been discovered that octopuses are able to distinguish between themselves and conspecifics through the use of chemoreception (Nesher et al., 2014) and vision (Tricarico et al., 2011). For instance, when presented with their own severed arms and those of conspecifics, octopuses exhibited differing behavioural responses, mediated by chemoreception. The test subjects were more likely to treat the arms of conspecifics as food objects than they did their own (Nesher et al., 2014). Octopuses are also able to recognize individual conspecifics, inferred from the increased tendency for aggressive behaviour toward other octopuses they had not previously encountered (Tricarico et al., 2011). Furthermore, octopuses’ individual recognition capacities also extend to humans. In a study by Anderson et al. (2010), identically dressed human handlers who would repeat respectively assigned behaviours regularly approached the octopuses over several days. Some of the handlers consistently offered the octopuses food, while the others would consistently poke them with a brush. Eventually, the octopuses exhibited markedly dissimilar behaviour toward the humans depending on whether they were food-bearing or obnoxious: whereas they approached the former, they tended to be more aggressive or avoidant toward the latter.

It may also be the case that if present, the sense of self in octopuses may go beyond simple self-other demarcation. This is suggested by observations that some octopus species, such as the mimic octopus (*Thaumoctopus mimicus*) rearrange their body outline, colouration, and texture and copy the locomotion techniques of non-conspecifics in order to imitate them (Hanlon and Messenger, 1996; Norman et al., 2001; Hanlon, 2007). This is usually done in potentially hazardous situations. For instance, when swimming across sand plains that offer little opportunities for hiding, octopuses may mimic flounders, which are less appealing to possible predators than octopuses are (Hanlon, 2007). When swimming in predator infested-waters, octopuses have also been observed to copy the posture and striped colouration of venomous lionfish in order to increase chances of safe passage (Norman et al., 2001). Octopuses are also known to pretend to be drifting seaweed (Hanlon and Messenger, 1996), especially when higher up in the water column. One technique used in this task is known as countershading, wherein certain parts of the body are darkened in order to resemble shadows cast by down-welling light. Together, these sophisticated forms of *crypsis* or disguise behaviour suggest that octopuses may be capable of awareness about how they appear from a third-person perspective, a capacity said to be dependent on consciousness and a sense of self.

Finally, the ability to sleep, which octopuses possess (Brown et al., 2006; Meisel et al., 2011; de Souza Medeiros et al., 2021), is also suggestive of consciousness. Along with attention and alertness, the awake state is typically regarded as an indication of a relatively high *level* of consciousness (in the sense of the intensity of conscious awareness), as it is “necessary for any meaningful responses to occur” (Blumenfeld, 2016, p. 4). In contrast, states such as sleep or coma are indicative of a low level of conscious awareness and arousal. As such, the capacity for sleep implies that an organism is able to alternate between states with high and low levels of consciousness (Siclari and Tononi, 2016). However, more detailed characteristics of consciousness in the organism in question are difficult to infer solely from the capacity to sleep.

These behaviours are among those suggestive of consciousness in octopuses. Importantly, they are capacities of the *intact* octopus, i.e., they emerge as the result of the complex interaction between the components of the nervous system. Consequently, investigations into consciousness in octopuses are based on a construal of the animal as a *coherent agent* (Godfrey-Smith, 2020), whose complex behaviour is the outcome of profound embodiment (Hochner, 2013) that evolved as a unique solution to the challenge of controlling a flexible body with immense sensory processing demands. Now, although we are unlikely to ever have complete knowledge about what it is like to be an octopus, attempting to understand consciousness in such creatures requires that we take a closer view at its arm nervous system, whose participation in cognition and behaviour is vital and indispensable.

## Arm-Based Consciousness?

We cannot say for certain, given knowledge about an animal’s nervous system or parts of it, what any conscious experience that arises from it might be like (Nagel, 1974; Chalmers, 1995). Although we can speculate what kinds of physiological states enter into a given creature’s consciousness (Morsella, 2005), this is difficult to ascertain from a third-person perspective. Thus, I will sidestep the task proper of consciousness attribution for now, and instead provide principled reasons for surmising that consciousness might exist in the arm nervous system.

The proposal that the octopus arm may be able to generate and support a local conscious field is motivated by the studies of Rowell (1963) and later on Sumbre et al. (2001) on amputated appendages, which demonstrate capacities for sensation and movement (or rudimentary action). If present, this arm-based consciousness would likely be *primary* consciousness, for which “a direct awareness of the world” (Barron and Klein, 2016, p. 4901) suffices. In the same vein, Peter Godfrey-Smith writes that minds (understood as equivalent to a conscious field) “have what we might call *characteristic interfaces*…that connect them with external objects and conditions. Sensing and action are the interfaces, and these mark the boundaries of a mind” (Godfrey-Smith, 2020, p. 290). Consequently, demarcating a unit that potentially generates a conscious field involves identifying constituent substrates that are responsible for sensation and action. As it is, the octopus arm is a structure that lends itself somewhat cleanly to such a demarcation task (at least in comparison to bisected human brains exhibiting the split-brain syndrome).

Noting that neither complexity of a process nor the need to integrate information from various sources in the nervous system is sufficient to guarantee a conscious field, Ezequiel Morsella (2005) proposed that the states that do enter into consciousness or are accompanied by phenomenal experience are those that are involved in the control of skeletal muscle. The reason for this is that the effectors can get in each other’s way when motor commands or action policies are not harmonised. He speculates that “conscious processes…mediate large-scale skeletomotor conflicts caused by structures in the brain with different agendas [and] behavioural tendencies…” (Morsella, 2005, p. 1010). As such, “phenomenal states could be considered as one of the mechanisms solving the problem of integrating processes in a largely parallel brain that must satisfy the demands of a skeletomotor system that can often express actions and goals only one at a time.” (Morsella, 2005, p. 1010). In other words, consciousness can help ensure that complex behaviour requiring the coordination of multiple effectors is carried out coherently, in part by interoceptively monitoring the effectors as they proceed with their movements. Likewise, Merker (2005) also suggests that the evolutionary emergence of consciousness was influenced by the need to distinguish between sensations caused by externally generated causes and internally generated ones, known as the *reafference problem*. Such a distinction is important, as it is prerequisite to determining whether a behavioural response to such signals is necessary or not, or what kind of response is warranted.

Importantly, these accounts, particularly Morsella’s, are largely vertebrate-based. However, assuming that the reason states involved in the control of skeletal muscles are conscious is not because they control skeletal muscles *per se*, but because of the need to ensure that effectors with limited motor capacities need to be harmonised in their movements, the same principle may apply to octopus arms. (If anything, such roles of consciousness might even be more beneficial or adaptive in octopuses given the virtually unlimited motor opportunities available to their arms). Consequently, these accounts can be recruited to help establish why, if ever, conscious experience evolved in octopus arms. The hydrostatic nature of octopus arm muscle entails that the stiffening of certain groups “provides skeletal support against which muscle contractions generate the movements” (Levy et al., 2017, p. 5). Thus, in a sense, the arm muscles are able to function as a sort of pseudo-skeleton that can be dissolved and reconstructed in different ways anywhere on the arm. Although there are mechanisms that prevent octopus arms with interfering with each other, such as chemical mechanisms in the skin that prevent the suckers of an arm from grasping another of the same animal’s other appendages (Nesher et al., 2014), and potential existence of gating mechanisms that direct arm extension commands to certain arms and not others (Zullo et al., 2009), these do not rule out the possibility of the presence of a conscious field in the octopus arm.

Although there is reason to believe that intact octopuses experience a single conscious field (Mather, 2021), the question remains as to where phenomenal experience in these animals is generated. In familiar models of consciousness, it is mostly the case that the CNS—particularly the brain—is the sole organ complex enough to be capable of generating a conscious field. This is not so in octopuses, whose arm nervous systems may be sophisticated enough to give rise to phenomenal consciousness, albeit rudimentary. If present, arm-based consciousness would consist of capacities for direct awareness about the world (Barron and Klein, 2016) and motor responses to active stimulation. Importantly, acknowledging that this is even a possibility entails a commitment to the view that consciousness comes in a spectrum of complexity, depending on its contents and the cognitive capacities it may engender or enable, ranging from the very simple to the highly sophisticated.

What I have suggested is that individual octopus arms may generated respective conscious fields, such that in an intact and anatomically normal octopus there may be eight of them. However, due to the profound interconnectedness of the arms into the network that is the arm nervous system, these fields may be experienced not disjointedly as a single field, which is then further incorporated into the conscious field generated by the brain. Now, whether the octopus experiences one single unified field or multiple distinct ones depends on how well they are *bound* together (O’Brien and Opie, 1998; Bayne, 2010) or fused into a conjoint phenomenal state.

If indeed octopuses experience a single, unified consciousness, then arm-based consciousness resembles the putative “two minds” of the split-brain syndrome: the multiplicity of conscious fields can only be manifested under conditions very different from the organism’s day-to-day experiences. In split-brain cases, this condition would be a specifically designed experimental setup; in octopuses it would be detachment of the arm. Without being subsumed under the octopus’s broader conscious field, the conscious field of the detached arm would simply be the conscious field of a detached octopus arm.

## Concluding Remarks

Being largely (and admittedly) speculative, the purpose of this manuscript is to motivate interest and set the stage for future research on the possibility that brains may not be the sole neural structure capable of generating consciousness. This is an important point in the study of animal minds: if we are to have a more comprehensive understanding of different types of creature consciousness, particularly those in invertebrates, we need to go beyond vertebrate-based assumptions about phenomenal experience. Among these assumptions are the notions that consciousness is by necessity unified, that there is only one conscious field per organism, and that only the CNS can generate conscious fields. There is no better case study in these possibilities than octopus arms and their idiosyncratic capacities.

## Data Availability Statement

The original contributions presented in the study are included in the article/supplementary material, further inquiries can be directed to the corresponding author.

## Author Contributions

The author confirms being the sole contributor of this work and has approved it for publication.

## Conflict of Interest

The author declares that the research was conducted in the absence of any commercial or financial relationships that could be construed as a potential conflict of interest.

## Publisher’s Note

All claims expressed in this article are solely those of the authors and do not necessarily represent those of their affiliated organizations, or those of the publisher, the editors and the reviewers. Any product that may be evaluated in this article, or claim that may be made by its manufacturer, is not guaranteed or endorsed by the publisher.

## Footnotes

1 An exception is *hectocotylisation*, or a modification for sexual purposes found in the third right arm of male octopuses.
